# Intravenous Injections to Replace Blood

**Published:** 1918-04

**Authors:** 


***Intravenous Injections to Replace Blood. A Report Presented to the Special Committee of the Medical Research Committee on Shock and Allied Conditions.*** By W. M. Bayliss, University Professor of General Physiology in University College. November 25th, 1917.

B. presents the results of experiments carried out to test the various solutions suggested as substitutes for blood in transfusion. Decerebrate cats were generally used, sometimes cats under urethane anesthesia, and occasionally dogs. About one-third to one-half of the calculated blood volume was first withdrawn from an artery, and an equal volume of the solution to be tested, warmed to 38°, injected into the external jugular vein. Care was taken that the temperature of the animal did not fall more than 1° C. in 3 hours.

*Solutions Containing Crystalloids Only.*

(*a*) Ringer's isotonic saline was found to have little value. The viscosity of saline solutions is much less than that of blood, so that the pressure never rises to its original height. Moreover, the fluid leaves the blood vessels very rapidly, causing edema of the tissues. Injection of Ringer's solution into the blood stream raises the filtration pressure, and also lowers by dilution the osmotic pressure due to the plasma colloids; it therefore increases the excess of loss by filtration.

(*b*) Hypertonic saline. Experiments made with 2 per cent. sodium chloride dissolved in tap water showed that at first water is attracted from the tissues; but this process only continues as long as the salt content of the blood exceeds that of the tissues. Owing to the free permeability of the capillaries to salts, the concentration of sodium chloride rapidly becomes equal on both sides; the osmotic force, opposing filtration due to blood pressure, ceases to be active; and the state of affairs is then similar to that with simple Ringer solution. There is, in fact, a progressive concentration of the blood, owing to loss of fluid. It seems that the period during which fluid is absorbed from the tissues by the hypertonic blood is very short; it is rapidly lost again, together with that injected.

(*c*) Calcium and Barium. Injection into the veins of 5 cc. of 1.1 per cent. calcium chloride causes a rapid rise of arterial pressure of about 20 mm.; this lasts for two or three minutes only and the pressure then returns to its previous level. The injection of an equivalent amount of sodium citrate after calcium produces no effect at all. One milligram of barium per kilo. caused a large rise of blood pressure but the succeeding fall was not delayed.

(*d*) Bicarbonate solutions. The existence of acidosis after loss of blood is a problem in course of investigation but must be reckoned with in the treatment of hemorrhage. Experiments seemed to show that bicarbonate combined with gum was not more effective than gum alone in simple hemorrhage.

(*e*) Glucose leaves the vessels less rapidly than salts, but, except in concentrations so great as to be dangerous to the heart, its advantages are small.

*Solutions Containing Colloids.*

The presence of a colloid having the osmotic pressure of those in the blood is as important as the existence of a proper viscosity in order to ensure that the rise be permanent and not merely momentary. For this purpose starch, dextrine, gelatine, and gum are the most useful substances, but a special study has been made of the last-named as it has all the properties of importance, and is free from certain objections which apply to the others.

Theoretically the best solution is between 6 and 7 per cent. of gumarabic in 0.9 per cent. sodium chloride: this has the same viscosity as *whole* blood and the same osmotic pressure as the colloids (proteins) of the plasma. The fact that limbs and other organs can be perfused for some hours with the solution without the production of edema shows that the osmotic pressure is high enough to prevent loss of fluid to the tissues. Clinically, it would probably be advisable not to use a solution stronger than 6 per cent., as, after hemorrhage, a 7 per cent. solution may be higher than that of the blood. As regards quantity, it would appear that, though 500 cc. has been the usual amount injected, more might be given with safety. The course of the blood pressure is the best guide. Experiments to test the possibility of anaphylaxis gave negative results.

*Preparation of Solutions.*

To make a 6 per cent. solution, dissolve the gum (the author used "Turkey Elect") in tap water on a water bath. Cool to room temperature. Add 2 per cent. sodium bicarbonate in powder. On shaking a precipitate of calcium carbonate separates. If possible, allow to stand for 24 hours; then filter through Chardin paper or centrifuge. Sterilize in *sealed* bottles in steam or in the autoclave, taking great care that no carbon dioxide escapes. The bottles with porcelain stopper and rubber washer, held by a wire clip, which are in common use for sterilized milk, have been found strong enough and suitable for the purpose. The whole bottle is warmed before injecting contents.

In cases of severe hemorrhage, when transfusion of blood is impossible, a certain proportion of washed blood corpuscles may be added to the gum solution with advantage; when this is done the percentage of gum should not be higher than five on account of the increase in viscosity.

